# Integrated single-cell and spatial transcriptomics reveal the differentiation drivers of gastric epithelial lineage progression

**DOI:** 10.3389/fimmu.2026.1712830

**Published:** 2026-02-09

**Authors:** Xuyu Chen, Xin Jiang, Siying Wang, Jianlei Xia, Wenjun Wang, Xinyu Fu, Ping Yukun, Zhuoqi Liu, Yaoyao Li, Min Zhang, Yanbing Ding

**Affiliations:** 1 Department of Gastroenterology, The Affiliated Hospital of Yangzhou University, Yangzhou University, Yangzhou, China; 2 Department of Oncology, The Second Affiliated Hospital of Nanjing Medical University, Nanjing, China; 3 Dalian Medical University, Dalian, Liaoning, China; 4 Department of Neurology, Northern Jiangsu People’s Hospital, Yangzhou, China

**Keywords:** epithelial differentiation, gastric cancer, Helicobacter pylori, intestinal metaplasia, UPP1

## Abstract

Gastric cancer (GC) develops through a sequence from chronic gastritis to intestinal metaplasia (IM) and carcinoma, with Helicobacter pylori (HP) as a key driver; however, the molecular mediators linking inflammation to malignant transformation remain unclear. We integrated single-cell RNA sequencing and spatial transcriptomics of gastric mucosal samples from atrophic gastritis, IM, and GC, including HP positive (+) and HP negative (-) cases, to map cellular heterogeneity, differentiation trajectories, and pathway activities. Our analyses revealed that IM epithelium represents a transitional state between normal and malignant epithelial lineages, characterized by enhanced WNT signaling that promotes neoplastic progression, whereas H. pylori–associated inflammation activates NF-κB signaling. Across analysis, UPP1 was consistently upregulated in malignant and H. pylori–positive epithelium, increasing along pseudotime toward cancer-like states. Spatial mapping and organoid experiments confirmed that UPP1-high cells had higher intestinal differentiation scores, while UPP1 knockout promoted IM-like morphology in WNT-depleted cultures. Clinically, UPP1 was elevated in GC versus normal tissues, correlated with advanced TNM stage, predicted poor survival, and was higher in HP^+^ tissues. Knockdown in GC cell lines reduced clonogenicity and migration. Collectively, these findings identify UPP1 as a key regulator of epithelial reprogramming and IM, linking H. pylori–driven inflammation with WNT-mediated differentiation, and highlight its potential as a prognostic biomarker and therapeutic target in GC.

## Introduction

Gastric cancer (GC) remains one of the leading causes of cancer-related mortality worldwide, with a particularly high incidence in world (1). The pathogenesis of GC is widely recognized as a multistep process, progressing from chronic gastritis to atrophic gastritis, intestinal metaplasia (IM), dysplasia, and finally invasive carcinoma, a sequence described as the Correa cascade (2, 3). Among these stages, IM is considered a key histological precursor of gastric cancer, especially for the intestinal-type GC. Epidemiological studies have shown that patients with IM have a significantly elevated risk of developing early gastric cancer, with cumulative incidence increasing with the extent and severity of IM (4, 5). Helicobacter pylori (HP) infection is a well-established initiator of this cascade, contributing to chronic inflammation, epithelial reprogramming, and genomic instability (6–8). Despite advances in endoscopic surveillance and HP eradication, the molecular drivers that govern the transition from IM to carcinoma remain incompletely understood.

At the cellular level, gastric epithelial homeostasis is maintained by a balance between stem cell renewal and lineage-specific differentiation (9, 10). Chronic inflammation disrupts this equilibrium by promoting excessive cytokine signaling, stem cell hyperproliferation, and lineage reprogramming, which together drive aberrant intestinal differentiation and increase susceptibility to IM and tumorigenesis. The WNT signaling pathway is a central regulator of gastric epithelial stem cell fate and has been implicated in the initiation and maintenance of IM (11, 12). Recent advances in single-cell RNA sequencing and spatial transcriptomics have enabled unprecedented resolution in mapping the cellular and molecular changes occurring during gastric carcinogenesis. However, integrative analyses that connect these high-dimensional datasets to functional validation in experimental models remain scarce.

In this study, we performed an integrative multi-omics analysis combining scRNA-seq, spatial transcriptomics, and functional assays to dissect the cellular trajectories, pathway dynamics, and molecular mediators involved in gastric epithelial transformation. By profiling gastric mucosal samples spanning atrophic gastritis, IM, and GC, with careful stratification by HP infection status, we identified IM-like cells as transitional intermediates between normal and malignant epithelium. Among differentially expressed genes, UPP1 (Uridine phosphorylase 1) emerged as a robust marker of IM and GC, with expression progressively increasing along pseudotime trajectories toward tumor-like states and correlating with HP infection. UPP1 is a key enzyme in pyrimidine salvage metabolism that catalyzes the phosphorolysis of uridine, thereby influencing nucleotide homeostasis, cellular stress responses, and metabolic adaptation. Increasing evidence links UPP1 to inflammation-driven metabolic remodeling and tumor progression in multiple cancers, suggesting it may play a functional role beyond serving as a marker. Functional validation using gastric organoid models demonstrated that UPP1 promotes intestinal-type differentiation, particularly under WNT-depleted conditions, and its loss alters organoid morphology toward IM-like phenotypes. Clinical analyses further confirmed that UPP1 is overexpressed in GC, associated with advanced TNM stage, and predicts poor survival. These findings establish UPP1 as a key mediator linking HP-driven inflammation and WNT-mediated epithelial reprogramming, offering a potential prognostic biomarker and therapeutic target for early intervention in gastric carcinogenesis.

## Materials and methods

### Patient recruitment and sample collection

Gastric tissues (normal, atrophic gastritis, intestinal metaplasia, gastric cancer) were obtained from the Affiliated Hospital of Yangzhou University with institutional review board approval and informed consent. Inclusion criteria were histopathological confirmation, no prior malignancy, no chemotherapy/radiotherapy, and consent to participate. Samples were collected during endoscopy or surgery.

### Gastric tissues single-cell RNA sequencing data processing

scRNA-seq data (GSE249874; 6 gastritis, 6 intestinal metaplasia, 6 gastric cancer; each with 3 HP–positive and 3 –negative) and gastric organoid scRNA-seq dataset GSE210991, comprising 26 gastric organoid samples (8 normal organoids and 18 intestinal metaplasia organoids) were processed in Seurat v5. Low-quality cells (high mitochondrial content) and doublets (DoubletFinder) were removed, and genes linked to mitochondrial function, heat shock, ribosomes, or dissociation artifacts were excluded. Data were integrated and batch-corrected with Harmony at both dataset and global levels for downstream analyses.

### Unsupervised dimensional reduction and clustering

Data were normalized (NormalizeData, Seurat v5), and the top 2000 variable genes were selected by VST (13). After scaling, PCA was performed, followed by Harmony integration, nearest-neighbor detection, and clustering (resolution 0.5). UMAP was used for visualization. Cluster markers were identified with FindAllMarkers, and cell types were annotated by integrating known lineage markers, FindAllMarkers results (14). Epithelial cells were classified into malignant and non-malignant populations using CNV inference (inferCNV). Immune cells served as reference populations, following published protocols.

### TCGA-STAD clinical association analysis

TCGA-STAD RNA-seq data and corresponding clinical information (T, N, M classification and overall stage) were obtained. UPP1 expression levels were analyzed in relation to tumor stage, and differences were visualized using ggplot2 with box and violin plots highlighting stage-wise comparisons.

### Non-negative matrix factorization

NMF was applied to malignant cells from each mouse organoid line with factorization ranks (K = 4–9). For each program, the top 50 genes were retained. Robust programs were defined by cross-K and cross-line overlaps, and redundant programs were removed based on gene sharing. Hierarchical clustering of non-redundant programs led to the identification of four meta-programs (MPs) consistently present across organoid lines.

### Cell culture

The GC cell lines AGS and HGC27, along with the normal gastric epithelial cell line GES-1, were obtained from the Nanjing Medical University laboratory. Prior to use, all cell lines were authenticated via short tandem repeat profiling and routinely screened for mycoplasma contamination. Cells were cultured in DMEM supplemented with 10% fetal bovine serum and 100 μg/ml penicillin/streptomycin. Cultures were maintained at 37 °C in a humidified incubator with 5% CO_2_.

### Multiplex immunofluorescence staining

Gastric epithelial organoids were stained for MUC2, CDX2, and DAPI. Sequential primary antibody incubation was followed by secondary antibodies with tyramide signal amplification (TSA) and microwave-based antigen retrieval between steps. Nuclei were counterstained with DAPI. Multispectral images were captured using the Mantra System (PerkinElmer) across 420–720 nm at 20 nm intervals, and autofluorescence was removed by spectral unmixing in InForm software (PerkinElmer).

### Cell proliferation and migration assays

Colony formation ability was assessed using 6-well plates. Briefly, cells were seeded at a density of 500 cells per well and cultured under standard conditions (37 °C, 5% CO_2_) for approximately 10–14 days until visible colonies formed. The culture medium was refreshed every 3 days. At the endpoint, colonies were washed twice with PBS, fixed with 4% paraformaldehyde for 20 min, and stained with 0.1% crystal violet solution for 30 min at room temperature. Excess dye was rinsed off with distilled water, and colonies containing >50 cells were counted under a light microscope. Quantification was performed in triplicate, and the colony formation efficiency was calculated as the ratio of colonies formed to the number of cells initially seeded. Cell migration was evaluated by a transwell chamber assay. In brief, 2 × 10^4^ cells suspended in 200 μL serum-free medium were seeded into the upper chamber of transwell inserts (8-μm pore size, Corning). The lower chamber was filled with 600 μL medium supplemented with 10% fetal bovine serum (FBS) to serve as a chemoattractant. After incubation for 24 h at 37 °C, non-migrated cells remaining on the upper surface of the membrane were gently removed with a cotton swab, while migrated cells on the underside of the membrane were fixed in 4% paraformaldehyde for 20 min and stained with 0.1% crystal violet. Stained cells were imaged and counted in at least five randomly selected microscopic fields per insert. Each experiment was performed in triplicate.

### Cell infection, plasmid construction, and transfection

AGS and HGC27 cells were transfected with control or target siRNAs (Proteinbio) or plasmids for UPP1 overexpression/shRNA knockdown (GeneCopoeia) using Lipofectamine 3000 (Invitrogen) per manufacturer’s protocol. Transfected cells were incubated for 48–72 h, and transfection efficiency was confirmed by RT-qPCR and Western blot. siRNA sequences are listed in the **Supplementary Data Sheet 1**.

### CRISPR–Cas9 knockout in gastric epithelial organoids

For UPP1 knockout, sgRNAs (sequences in **Supplementary Data Sheet 1**) were cloned into pSpCas9(BB)-2A-Puro (PX459, Addgene #48139). Gastric epithelial organoids were dissociated into single cells, transfected with 1 µg plasmid using Lipofectamine 3000 (Invitrogen), and selected in organoid medium containing 0.3 mg/mL puromycin. Surviving clones were expanded in Matrigel domes, and knockout was confirmed by Sanger sequencing. For sequential TP53/UPP1 knockout, TP53-null organoids were first generated by transfecting an all-in-one TP53 sgRNA–Cas9 construct, followed by nutlin-3 selection to enrich TP53-null clones, which were clonally expanded and sequence-verified. TP53-null organoids were then transduced with a lentiviral Cas9 construct conferring blasticidin resistance, followed by infection with a lentivirus carrying an UPP1 sgRNA and puromycin resistance gene. After puromycin selection, sgRNA-positive single cells were sorted into 96-well plates for clonal expansion. UPP1 loss was confirmed by sequencing.

### Gastric organoid culture

Fresh gastric tissues from gastrectomy patients were processed under ethical approval and the Declaration of Helsinki. Tissues were minced, washed in cold PBS, digested in collagenase IV solution (advanced DMEM/F12 with 1 mg/mL collagenase IV, 1× P/S) for 40 min at 37 °C, and mechanically dissociated. After PBS washes and centrifugation, pellets were resuspended in Matrigel (50ul/well, 24-well plate). Once solidified, 500ul gastric organoid medium (advanced DMEM/F12 supplemented with GlutaMax, HEPES, P/S, B27, N2, R-spondin-1, Noggin, EGF, FGF10, HGF, N-acetylcysteine, Gastrin, Nicotinamide, Forskolin, A83-01, Y-27632, Dexamethasone, and Primocin) was added. Organoids were cultured at 37 °C, 5% CO_2_, and passaged every 7–10 days.

### Quantitative real-time PCR

Total RNA was extracted with TRIzol and quantified using NanoDrop. cDNA was synthesized with HiScript III All-in-One RT SuperMix (Vazyme), and RT-qPCR was performed with SYBR Green Master Mix using gene-specific primers (**Supplementary Material**) and GAPDH as reference. Cycling conditions were 95 °C for 2 min, followed by 40 cycles of 95 °C for 15 s and 60 °C for 1 min. Relative expression was calculated by the 2^–ΔΔCt^ method from triplicate experiments.

### Western blot analysis

Tissues were lysed in RIPA buffer (Beyotime) containing protease and phosphatase inhibitors, and protein concentration was determined by BCA assay (Thermo Fisher). Equal amounts of protein were separated by SDS–PAGE, transferred to nitrocellulose membranes, and blocked with 5% non-fat milk in TBST. Membranes were incubated overnight at 4 °C with anti-UPP1 and anti-ACTIN antibodies, followed by HRP-conjugated secondary antibodies. Bands were visualized using ECL (Bio-Rad) and quantified with ImageJ.

### Spatial transcriptomic data analysis

Raw reads were processed and mapped with Spaceranger. Data were normalized using LogVMR and filtered by Space Ranger quality control. Dimensionality reduction (PCA, 30 PCs) and clustering (resolution 0.5) were performed in Seurat, and spatial feature plots were generated with SpatialFeaturePlot. Cell type abundance was estimated using two complementary approaches: CARD deconvolution based on signature genes for tumor, epithelial, and immune subsets (top 30 markers from FindAllMarkers), and SpaCET for spatially informed deconvolution. Pathway activity scores for each spatial spot were computed using decoupleR, enabling integration of transcription factor and pathway activity inference with spatial context.

### Cell differentiation analysis

Cellular differentiation potential was inferred using CytoTRACE, with higher scores indicating less differentiated cell states. For trajectory analysis, cells were ordered along pseudotime using monocle3 based on the top variable genes from Seurat. The root state was defined according to the CytoTRACE-predicted least differentiated population, and branch-dependent gene expression dynamics were visualized to characterize lineage progression.

### Online database analysis

UPP1 mRNA expression in STAD was analyzed using GEPIA (TCGA and GTEx integration). Protein expression and IHC images were obtained from the Human Protein Atlas. Prognostic value was assessed with Kaplan–Meier Plotter by stratifying patients into high- and low-expression groups.

### Statistical analysis

Data were analyzed in R (v4.4.2) and GraphPad Prism (v9). *In vitro* experiments were performed in triplicate. Student’s t-test was used for normally distributed data, and non-parametric tests for non-Gaussian data. p < 0.05 was considered statistically significant.

## Results

### Single-cell transcriptomic landscape across gastric lesion progression and Helicobacter pylori infection status

**Figure 1:**
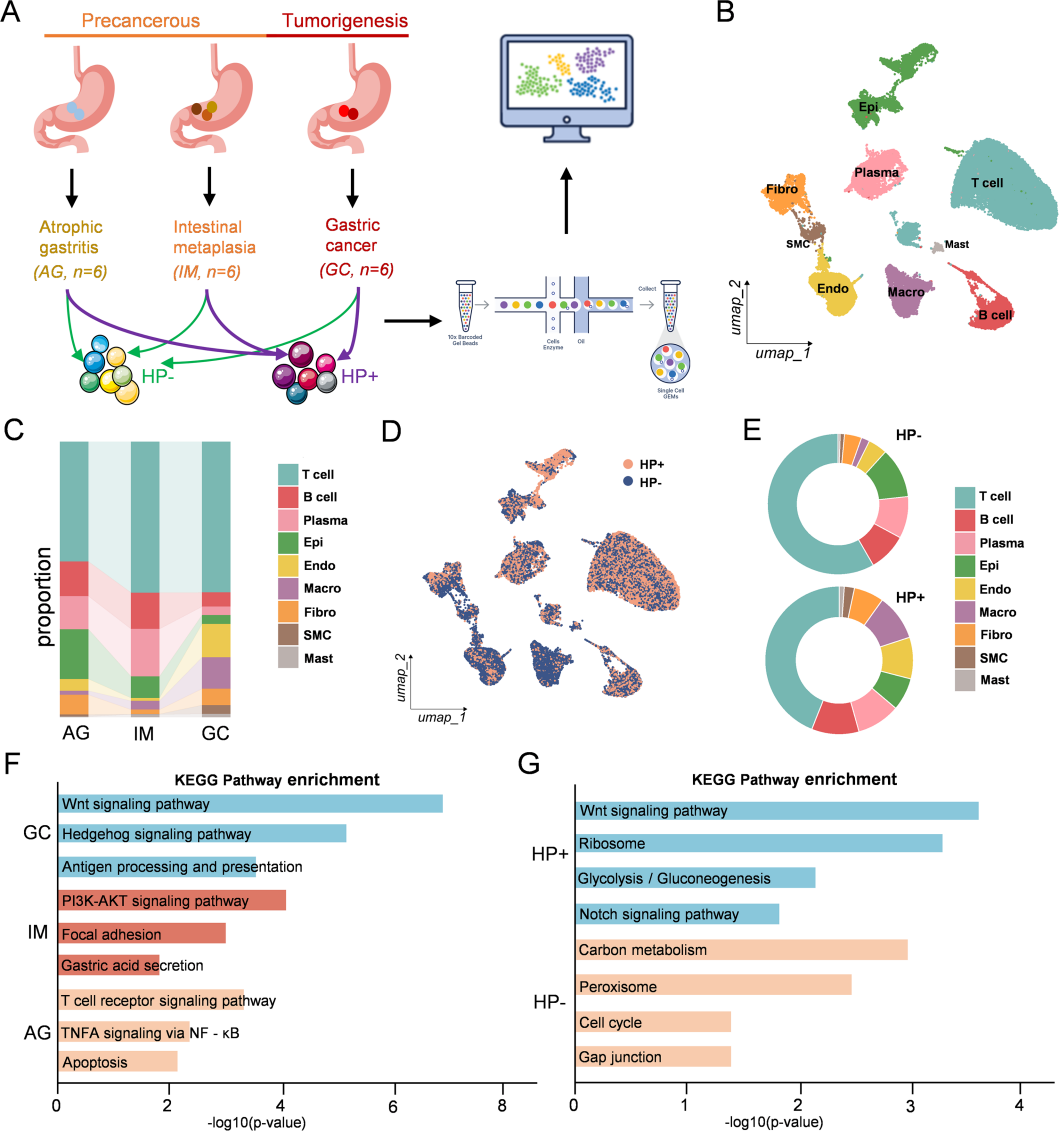
Single-cell transcriptomic landscape across gastric lesion progression and Helicobacter pylori infection status. **(A)** Schematic workflow of sample collection and scRNA-seq analysis. Eighteen human gastric mucosal samples, including atrophic gastritis (AG, n=6), intestinal metaplasia (IM, n=6), and gastric cancer (GC, n=6), were obtained, with equal numbers of **(H)** pylori (HP) positive and negative cases. **(B)** UMAP visualization of major cell types annotated by canonical marker genes, including epithelial cells, fibroblasts, endothelial cells, smooth muscle cells, macrophages, mast cells, T cells, B cells, and plasma cells. **(C)** Stacked bar plots showing cell-type composition across disease stages. **(D)** UMAP plots of HP^+^ vs. HP^-^ samples. **(E)** Donut plots showing cell-type proportions in HP^+^ and HP^-^ samples. **(F)** KEGG enrichment of upregulated genes in GC, IM, and AG. **(G)** KEGG enrichment of upregulated genes in HP^+^ and HP^-^ groups.

To investigate cellular heterogeneity and molecular dynamics during gastric carcinogenesis, we performed single-cell RNA anslysis on 18 human gastric mucosal samples, including 6 atrophic gastritis (AG), 6 IM, and 6 GC tissues (**Figure 1A**). clinical information summarized in **Supplementary Table S1**. For each pathological stage, equal numbers of Helicobacter pylori-positive (HP^+^, n = 3) and HP-negative (HP^-^, n=3) samples were included. After quality control and integration, unsupervised clustering identified major cell types including epithelial cells, fibroblasts, endothelial cells, smooth muscle cells, macrophages, mast cells, T cells, B cells, and plasma cells, based on canonical marker genes (**Figure 1B**). Cell-type annotations were determined based on canonical marker genes, which are summarized in **Supplementary Table S2**. Cell-type composition varied across the disease spectrum. (**Figure 1C**). When stratified by *HP* status, UMAP visualization revealed no obvious segregation of HP^+^ and HP^-^ cells (**Figure 1D**). Interestingly, when comparing the cellular composition across the three progressive pathological stages (AG, IM, and GC), we observed a gradual decline in the proportion of epithelial cells as the disease advanced toward carcinoma. In parallel, macrophages were markedly increased in GC compared with AG and IM. Increase in endothelial cells which is also perfectly correlated between the progressive pathological stages and HP^-^ samples. Moreover, compared with uninfected tissues, HP-infected samples contained a relatively lower proportion of epithelial cells but a higher proportion of macrophages, mirroring the compositional changes observed across AG, IM, and GC, thereby reflecting the carcinogenic role of *HP*. These findings reinforce the evidence that gastric tumorigenesis is accompanied by significant shifts in cellular composition and highlight the dynamic remodeling of the tumor microenvironment (**Figure 1E**). We next performed KEGG pathway enrichment analyses on differentially expressed genes (DEGs) across disease stages and infection status. During GC progression, genes upregulated in GC samples were enriched for the Wnt and Hedgehog signaling pathways, while IM samples were enriched for pathways such as focal adhesion and gastric acid secretion. In contrast, AG samples showed enrichment in TNF signaling and apoptosis-related pathways (**Figure 1F**). Moreover, genes enriched in HP^+^ samples were associated with Wnt signaling, ribosome function, and glycolysis, while HP^-^ samples showed enrichment in cell cycle, peroxisome, and gap junction pathways (**Figure 1G**). As Wnt signaling is a differentiation-associated pathway that emerges both during malignant transformation and in the context of HP infection, this underscores its pivotal role in the dynamic evolution of epithelial carcinogenesis.

### Pseudotime trajectory reveals an epithelial transition with elevated UPP1 expression

**Figure 2:**
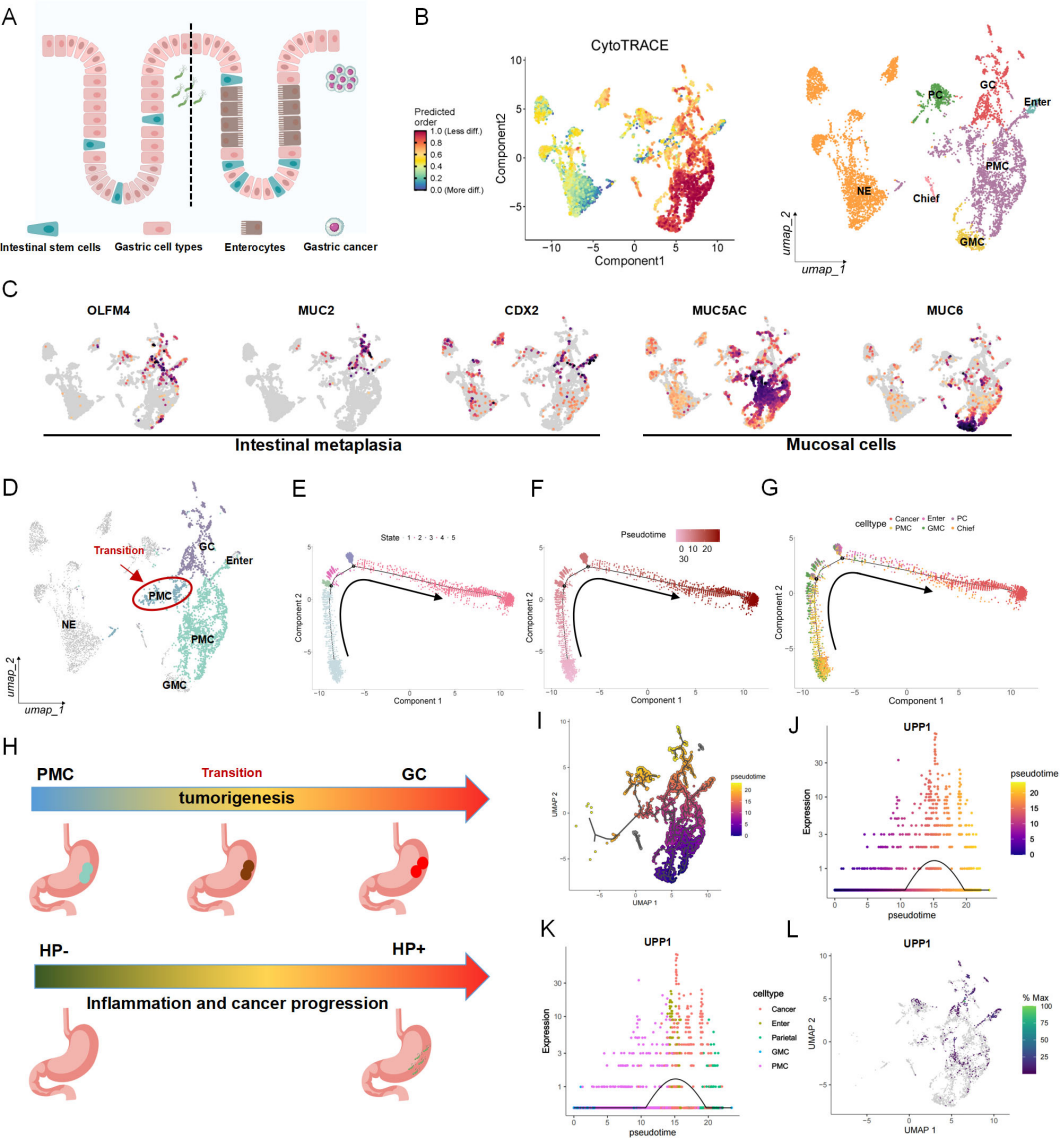
Pseudotime trajectory reveals a normal-to-cancer epithelial transition with elevated UPP1 expression. **(A)** Diagram of gastric epithelial lineages, including intestinal metaplasia and gastric cancer. **(B)** CytoTRACE analysis showing differentiation states of epithelial subtypes. **(C)** UMAP plots with epithelial subtype annotations based on canonical markers, including intestinal markers (OLFM4, MUC2, CDX2) and mucous cell markers (MUC5AC, MUC6). **(D)** Identification of highly differentiated cell population as a transitional population between normal and malignant epithelial cells. **(E–G)** Pseudotime trajectories showing progression from non-neoplastic to cancer epithelial cells. **(H)** Schematic summarizing GC transition and HP-related inflammation–cancer progression. **(I, J)** UPP1 expression along pseudotime in tumorigenesis differentiation. **(K)** UPP1 expression trends in different epithelial subtypes. **(L)** UMAP visualization of UPP1 expression distribution.

To further dissect epithelial lineage progression during AG, IM and GC development, we constructed a differentiation framework encompassing gastric gland epithelial subtypes (**Figure 2A**). First, we performed subclustering of epithelial populations and further assessed their differentiation potential using CytoTRACE. In the visualization, deeper red indicates lower differentiation status, whereas lighter colors represent more differentiated states (**Figure 2B**). In parallel, epithelial cells were annotated based on canonical marker genes, all of which are summarized in **Supplementary Table S2**. Gastric epithelial cells were mainly classified into pit mucous cells (PMC), neck mucous cells, entercytes, chief cells, parietal cells, neuroendocrine cells, and cancer cells. Tumor cells exhibited extensive copy number variations and simultaneously showed high expression of CEACAM5 and CEACAM6. Marker gene expression confirmed the identity of intestinal metaplasia-related genes such as OLFM4, MUC2, and CDX2, and mucous cell markers MUC5AC and MUC6, reinforcing the cellular composition associated with metaplastic and neoplastic transformation (**Figure 2C**). Based on differentiation status, we identified a subset of cells within the PMC population that exhibited a markedly higher degree of differentiation, displaying a tendency toward both intestinal metaplasia and tumor-like phenotypes. We designated this highly differentiated subset of PMC as “transition cells,” which may represent an intermediate state in epithelial lineage evolution during gastric carcinogenesis(**Figure 2D**). Pseudotime analysis reconstructed the entire differentiation trajectory from normal epithelium to intestinal metaplasia and ultimately to tumor states. Notably, a subset of PMC cells was positioned along the same pseudotime axis as intestinal metaplasia, further supporting the notion that the transition cells represent an intermediate state, in which highly stem-like epithelial cells dynamically shift from a normal phenotype toward intestinal metaplasia during gastric carcinogenesis (**Figures 2E–G**). To visualize and summarize these findings, we constructed two schematic models: the first depicts the epithelial transition from PMC to transition cells to GC cells, and the second illustrates the impact of HP infection in promoting inflammation-associated cancer progression (**Figure 2H**). To identify key regulators involved in epithelial differentiation during carcinogenesis, we screened genes that were dynamically altered along the pseudotime trajectory (**Figure 2I**) as well as those upregulated in the context of Helicobacter pylori infection. We found that UPP1 was markedly elevated during both intestinal metaplasia and malignant transformation, and was also significantly upregulated in HP-infected samples (**Figure 2J**). Therefore, we selected UPP1 as the focus of our subsequent investigation.This trend was further confirmed by plotting expression across pseudotime and epithelial subtypes (**Figure 2K**), where UPP1 expression was specifically elevated in metaplastic and malignant epithelial clusters. UMAP visualization showed that UPP1 expression was spatially enriched in regions corresponding to IM and GC epithelial populations, with minimal expression in normal epithelium (**Figure 2L**), highlighting its potential role as a marker of intestinal-type tumorigenic progression.

### Spatial transcriptomic profiling delineates distinct gastric regions and cellular composition

**Figure 3:**
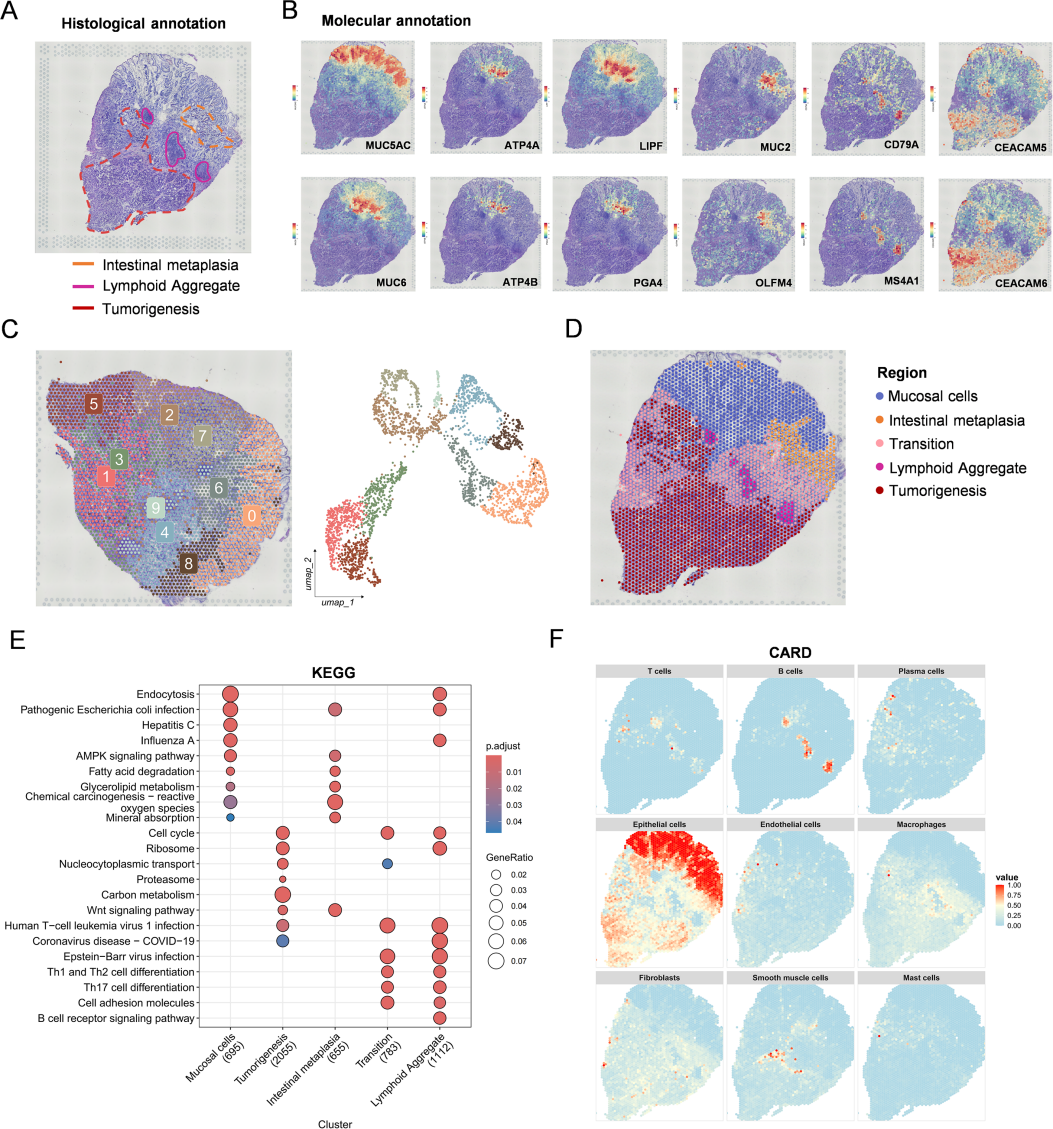
Spatial transcriptomic profiling delineates distinct gastric regions and cellular composition. **(A)** Histological annotation of gastric cancer tissue identifying mucosal cells, intestinal metaplasia, transition zones, lymphoid aggregates, and tumorigenesis regions. **(B)** Spatial expression of key marker genes (e.g., MUC5AC, MUC6, MUC2, OLFM4, CEACAM5, CEACAM6, CD79A, MS4A1). **(C)** Spot-level clustering results in the spatial tissue. **(D)** Region merging to define five major histological areas. **(E)** KEGG pathway enrichment of region-specific DEGs. **(F)** CARD deconvolution showing cell-type distribution based on scRNA-seq reference.

To spatially resolve the transcriptional heterogeneity of gastric cancer tissues, we performed spatial transcriptomics on a representative gastric cancer sample. We also conducted preliminary analyses across multiple spatial samples and, after comparison, selected the sample that contained both well-defined epithelial and intestinal metaplasia regions for further investigation (**Supplementary Figure S1**). Based on histological evaluation by experienced pathologists, five major regions were annotated: normal mucosal epithelium, intestinal metaplasia, transitional zones, lymphoid aggregates, and areas of tumorigenesis. These regions were outlined and color-coded to guide downstream spatial analysis (**Figure 3A**). Molecular annotation further validated histological segmentation. Spatial expression of key marker genes revealed clear regional specificity (**Figure 3B**). For instance, MUC5AC and MUC6 marked gastric epithelial lineages, while MUC2, OLFM4, and CDX2 were predominantly enriched in the intestinal metaplasia region. In contrast, tumor regions displayed strong expression of CEACAM5 and CEACAM6, canonical markers of gastric malignancy. The spatial localization of immune-related genes such as CD79A, MS4A1 (B cells), and LIPF also aligned with lymphoid aggregate regions, reinforcing the accuracy of molecular demarcation. Unsupervised clustering of spatial transcriptomic spots yielded ten distinct clusters, each exhibiting unique spatial distributions corresponding to anatomically defined regions. Integration with UMAP embedding enabled mapping of transcriptionally similar cell populations across modalities (**Figure 3C**). To classify region-level comparisons, clusters were manually merged into five biologically meaningful regions: mucosal cells, intestinal metaplasia, transition zones, lymphoid aggregates, and tumorigenesis (**Figure 3D**). We then performed KEGG enrichment analysis on the region-specific differentially expressed genes. Tumorigenic areas were enriched in pathways related to cell cycle, proteasome activity, and chemical carcinogenesis (**Figure 3E**), consistent with active proliferation and malignant transformation. The intestinal metaplasia region showed upregulation of fatty acid degradation and mineral absorption pathways, while lymphoid aggregates were enriched in immune-related processes such as B-cell receptor signaling and T-helper cell differentiation. To infer cellular composition at high resolution, we applied CARD analysis, leveraging our matched scRNA-seq reference. The enrichment of immune-related KEGG pathways in transition cells likely reflects their close spatial association with lymphoid aggregates and exposure to a cytokine-rich inflammatory microenvironment, leading to activation of immune-responsive signaling programs. The deconvolution revealed a spatially organized distribution of major gastric cell types (**Figure 3F**). Furthermore, we applied the SpaCET algorithm to refine spatial identification of tumor regions. Notably, malignant cell state A corresponded to the tumor region, while state B aligned with the transition zone, thereby validating the accuracy of our regional classification (**Supplementary Figure S2**). Epithelial cells were predominantly located in mucosal and tumor regions, whereas plasma and B cells localized to lymphoid aggregates. The spatial distribution of endothelial cells, fibroblasts, and macrophages also aligned with tissue structure, further confirming the consistency between pathological annotation, marker gene expression, and cellular mapping.

### Spatial and functional characterization of WNT signaling dynamics during gastric epithelial transformation

**Figure 4:**
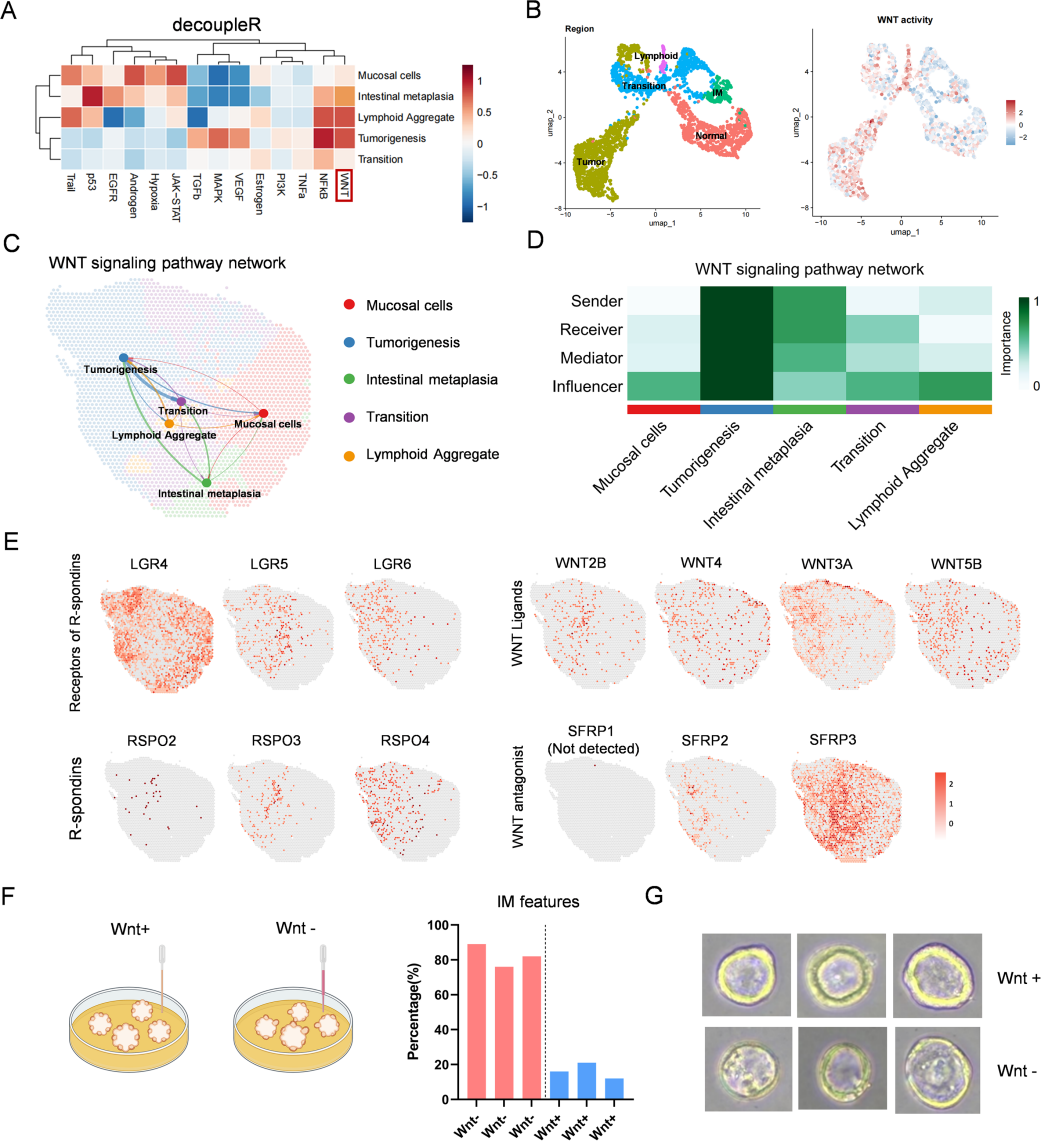
WNT signaling orchestrates epithelial differentiation and intestinal metaplasia development. **(A)** Pathway activity scores (decoupleR) showing NF-κB and WNT signaling activation across pathological regions. **(B)** WNT pathway scoring across spatial clusters. **(C)** WNT signaling communication network among histological regions. **(D)** Heatmap showing WNT signaling roles (sender, receiver, mediator, influencer) by region. **(E)** Spatial expression of WNT receptors (LGR4/5/6), ligands (WNT2B, WNT3A, WNT4, WNT5B), R-spondins (RSPO2/3/4), and antagonists (SFRP1/2/3). **(F)** Organoid culture experiments showing increased intestinal metaplasia features under WNT-depleted conditions. **(G)** Representative brightfield images of organoids under WNT^+^ and WNT^-^ conditions.

In both single-cell and spatial transcriptomic datasets, WNT signaling emerged as a recurrently activated pathway associated with epithelial differentiation. Given its well-established role in regulating stem cell fate and lineage specification, we further investigated the spatial and functional dynamics of WNT signaling in the context of gastric epithelial progression from normal mucosa to intestinal metaplasia (IM) and tumorigenesis. We first employed the decoupleR framework to score the activity of key signaling pathways across histologically defined regions (**Figure 4A**). Among the most prominently activated pathways were NF-κB and WNT, both of which displayed strong activation in intestinal metaplasia and tumorigenesis. While NF-κB signaling was primarily enriched in response to inflammation in normal mucosa, likely contributing to inflammatory-induced epithelial reprogramming, WNT signaling was specifically elevated along the differentiation trajectory toward metaplastic and neoplastic fates—suggesting its role in guiding lineage transition downstream of stem cell activation. WNT signaling primarily supports epithelial proliferation and stem-like states, whereas attenuation of WNT activity facilitates epithelial differentiation, including metaplastic programs, in a context-dependent manner. To evaluate regional differences in WNT activity, we clustered spatial transcriptomic spots according to histological status and visualized WNT signaling scores across the tissue sections (**Figure 4B**). WNT activity was highest in IM, transitional zones, and tumorigenesis regions, whereas scores were lower in normal epithelial zones, indicating spatial specificity of WNT-driven reprogramming. We then conducted spatial communication analysis focused on WNT pathway interactions among the annotated regions (**Figure 4C**). WNT signaling exhibited higher levels of inter-regional crosstalk, particularly among IM and tumorigenesis zones, indicating strong paracrine and autocrine signaling networks during disease progression. Quantitative network modeling suggested that intestinal metaplasia regions displayed enhanced signaling activity as senders and mediators, while tumorigenic regions showed increased incoming signaling interactions (**Figure 4D**). This directional pattern highlights a progressive WNT-driven communication axis promoting malignant transformation. To dissect the molecular components of WNT signaling, we spatially mapped the expression of ligands (WNT2B, WNT3A, WNT4, WNT5B), receptors (LGR4/5/6), co-activators (RSPO2/3/4), and antagonists (SFRP1/2/3) across the tissue. Notably, LGR4, WNT4, WNT5B, and SFRP3 were broadly distributed across all regions, suggesting a constitutive role in baseline WNT signaling (15). In contrast, genes such as LGR5/6, WNT3A, and RSPO2/3/4 were specifically enriched in IM and tumorigenesis zones, indicating the presence of distinct WNT sub-pathway modules that may mediate region-specific epithelial responses to external cues (**Figure 4E**). To experimentally validate the role of WNT signaling in epithelial lineage determination, we established a gastric organoid differentiation model. Wnt3a and R-spondin are key components of gastric epithelial organoid culture medium that critically regulate epithelial differentiation. We therefore plan to modulate Wnt3a to adjust Wnt pathway activity and its influence on epithelial differentiation, thereby constructing a gastric epithelial differentiation model in which Wnt3a functions as a molecular switch. Human gastric epithelial organoids were cultured in the presence (Wnt+) or absence (Wnt-) of WNT factors. (**Figure 4F**). After 4 weeks, morphological assessments of 100 organoids per group revealed that Wnt- conditions strongly promoted intestinal metaplasia-like features, characterized by edge thickening of the organoid epithelium, vacuolization within epithelial structures, and increased morphological heterogeneity. In contrast, organoids cultured with WNT supplementation maintained a proliferative, undifferentiated epithelial morphology. Representative brightfield images confirmed that Wnt-depleted organoids exhibited more differentiated structures (**Figure 4G**), reinforcing the important role of WNT signaling on metaplastic differentiation. Collectively, these findings underscore a pivotal role for WNT signaling in mediating the transition from gastric stem-like epithelial cells to metaplastic and neoplastic lineages, acting both spatially and functionally to coordinate epithelial fate during gastric carcinogenesis.

### UPP1 and TP53 cooperatively regulate intestinal metaplasia development in human gastric organoids

**Figure 5:**
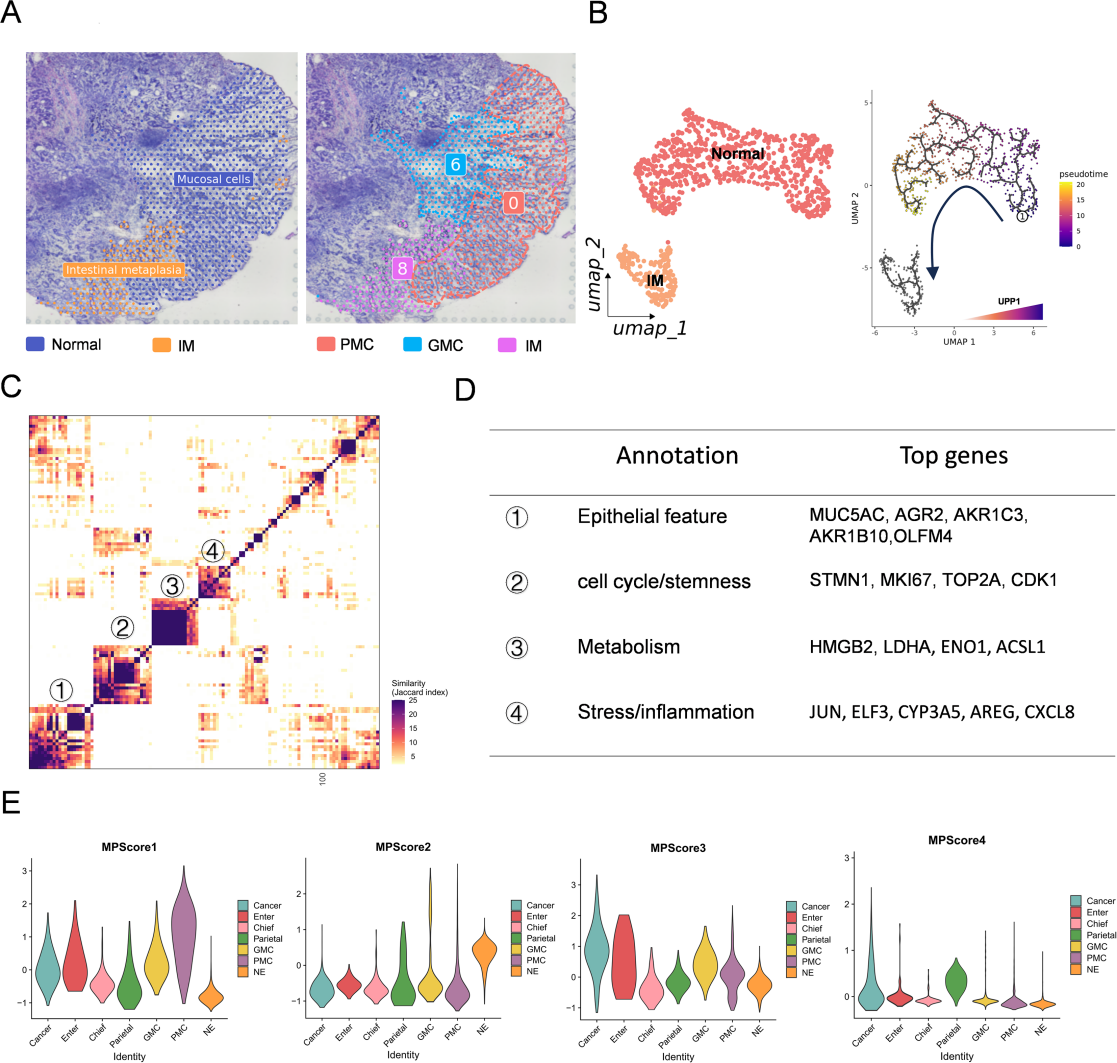
UPP1 regulate intestinal metaplasia development in human gastric organoids. **(A)** Spatial annotation of normal and IM regions, showing IM proximity to surface mucous cells. **(B)** Pseudotime trajectory from normal epithelium to IM, with UPP1 expression increasing along the path. **(C)** NMF clustering of gastric epithelial cells identifying four major meta-programs (MPs). **(D)** Definition of MPs by their top 50 genes, corresponding to epithelial feature, cell cycle/stemness, metabolism and stress/inflammation. **(E)** MP activity scoring across epithelial cells, showing that metabolic and stress-response programs are strongly enriched in IM and tumor epithelial populations.

To experimentally validate the molecular regulation of intestinal metaplasia (IM) observed in single-cell and spatial datasets, we established a primary human gastric organoid culture system derived from gastric corpus tissues. Tissues were enzymatically digested and embedded in Matrigel droplets for long-term expansion and differentiation in 6-well plates. Histological annotation of spatial transcriptomic sections revealed that IM regions were predominantly adjacent to surface mucous cells, whereas neck mucous cells resided more proximally to the gland base (**Figure 5A**). This spatial relationship suggested that stem-like gastric epithelial cells may serve as the cellular origin for IM development. To reconstruct the differentiation process, we extracted ST spots corresponding to normal and IM regions and performed pseudotime trajectory analysis using Monocle3 (**Figure 5B**). The trajectory revealed a progressive transition from normal mucosal cells to metaplastic lineages. Importantly, UPP1 expression gradually increased along the pseudotime axis, mirroring the trend previously observed in single-cell datasets, further supporting its role in metaplastic transformation. To further characterize transcriptional heterogeneity within gastric epithelial cells, we performed non-negative matrix factorization clustering and identified four major meta-programs (MPs) (**Figure 5C**). Each MP was defined by its top 50 weighted genes (**Figure 5D**), which captured distinct biological processes: epithelial feature, cell cycle/stemness, metabolism and stress/inflammation. We next scored the activity of these four MPs across epithelial subpopulations and disease stages (**Figure 5E**). Notably, the metabolic and stress-response MPs were significantly enriched in IM and tumor epithelial cells compared with normal epithelium, indicating their critical roles in lineage reprogramming and malignant transformation. These findings underscore the importance of metabolic remodeling and stress adaptation in gastric carcinogenesis and highlight UPP1, a pyrimidine metabolism enzyme, as a representative factor bridging metabolic reprogramming and epithelial progression. Knockout of TP53 enables gastric epithelial organoids to undergo long-term passaging (16). Given the frequent inactivation of TP53 in gastric cancer and the upregulation of UPP1 during metaplasia, we hypothesized that these two factors may cooperatively promote IM. To dissect the functional role of UPP1, we generated TP53/UPP1 double-knockout (DKO) gastric organoids using CRISPR-Cas9 and established UPP1-overexpression organoids through lentiviral delivery, enabling parallel evaluation of loss- and gain-of-function phenotypes (**Supplementary Figure S3**). Compared to wild-type organoids, which maintained a compact and spherical morphology, DKO organoids exhibited hallmarks of intestinal metaplasia, including vacuolization, morphological heterogeneity, and luminal expansion—recapitulating *in vivo* features of early metaplastic transformation. To further confirm this phenotype at the molecular level, we performed immunofluorescence staining for canonical IM markers MUC2 and CDX2. While WT organoids displayed minimal or no expression of these markers, DKO organoids showed robust upregulation of both MUC2 and CDX2, providing strong evidence that combined loss of TP53 and UPP1 drives a transcriptional program associated with intestinal differentiation. Collectively, these findings functionally validate the involvement of TP53 and UPP1 in promoting IM, and support a model in which WNT signaling, epithelial cell fate regulators, and tumor suppressor dysfunction converge to orchestrate gastric epithelial metaplasia and early neoplastic transformation.

### UPP1 is overexpressed in gastric cancer and promotes tumor proliferation and invasion

**Figure 6:**
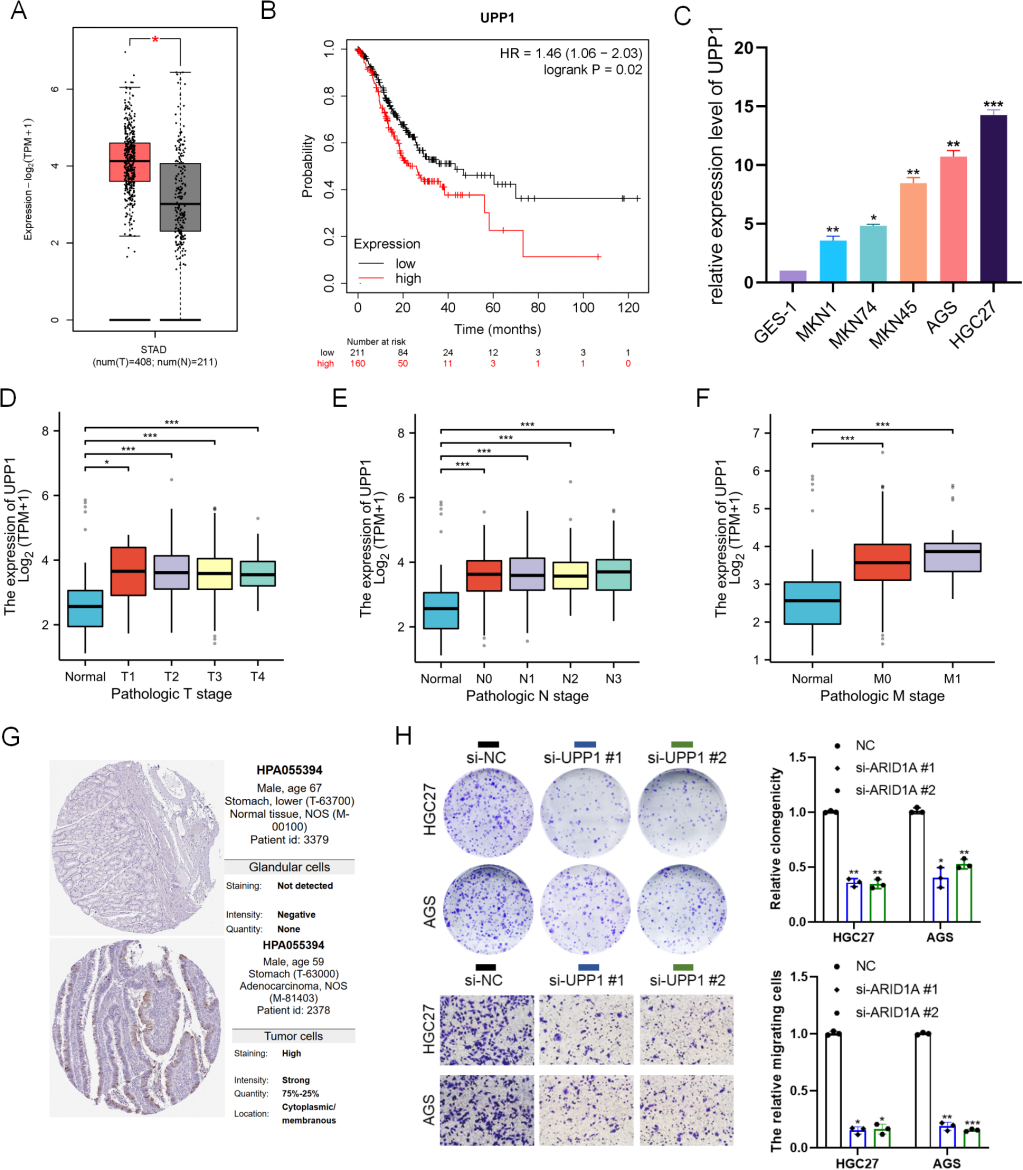
UPP1 is overexpressed in gastric cancer and promotes tumor proliferation and invasion. **(A)** TCGA-STAD analysis showing higher UPP1 expression in gastric cancer tissues compared with normal tissues. **(B)** Kaplan–Meier survival curve stratified by UPP1 expression level. **(C)** UPP1 mRNA levels in normal gastric epithelial cells (GES-1) and gastric cancer cell lines. **(D–F)** Association between UPP1 expression and pathological TNM stage. **(G)** Immunohistochemistry from Human Protein Atlas showing low UPP1 in normal tissue and high expression in gastric cancer. **(H)** Functional assays in AGS and HGC27 cells showing reduced clonogenicity and migration after UPP1 knockdown. *p < 0.05; **p < 0.01; ***p < 0.001.

To further validate the clinical relevance of UPP1 gastric tumorigenesis, we first analyzed its expression in the TCGA-STAD cohort, which includes 408 GC tumor samples and 211 normal gastric tissues. UPP1 was significantly upregulated in tumor tissues compared to normals (**Figure 6A**). Kaplan–Meier survival analysis showed that high UPP1 expression was associated with poor overall survival in gastric cancer patients (**Figure 6B**). We next assessed UPP1 mRNA levels in a panel of gastric cell lines. Compared to the normal gastric epithelial cell line GES-1, UPP1 expression was markedly elevated in multiple gastric cancer cell lines, including MKN1, MKN74, MKN45, AGS, and HGC27, with the highest levels observed in AGS and HGC27 cells (**Figure 6C**). Furthermore, correlation analyses revealed that UPP1 expression significantly increased with tumor progression, showing higher levels in advanced T stages (**Figure 6D**), N stages (**Figure 6E**), and M stages (**Figure 6F**), supporting a strong link between UPP1 and clinical malignancy. Consistent with IHC images from the Human Protein Atlas confirmed that UPP1 was barely detectable in normal gastric tissues but strongly expressed in gastric adenocarcinoma specimens, particularly in the cytoplasmic and membranous compartments of tumor cells (**Figure 6G**, left panel). To functionally evaluate the oncogenic role of UPP1, we performed gene function assays in AGS and HGC27 cells using two independent siRNAs. Clonogenic assays revealed that knockdown of UPP1 significantly impaired the colony-forming ability of both cell lines (**Figure 6H**). Moreover, transwell migration assays demonstrated a marked reduction in cell motility upon UPP1 silencing (**Figure 6H**), indicating that UPP1 facilitates both proliferation and invasive behavior in gastric cancer cells. These data demonstrate that UPP1 is a clinically relevant oncogene in gastric cancer, contributing to tumor progression and serving as a potential prognostic and therapeutic target.

### UPP1 promotes intestinal metaplasia in gastric epithelial organoid models

**Figure 7:**
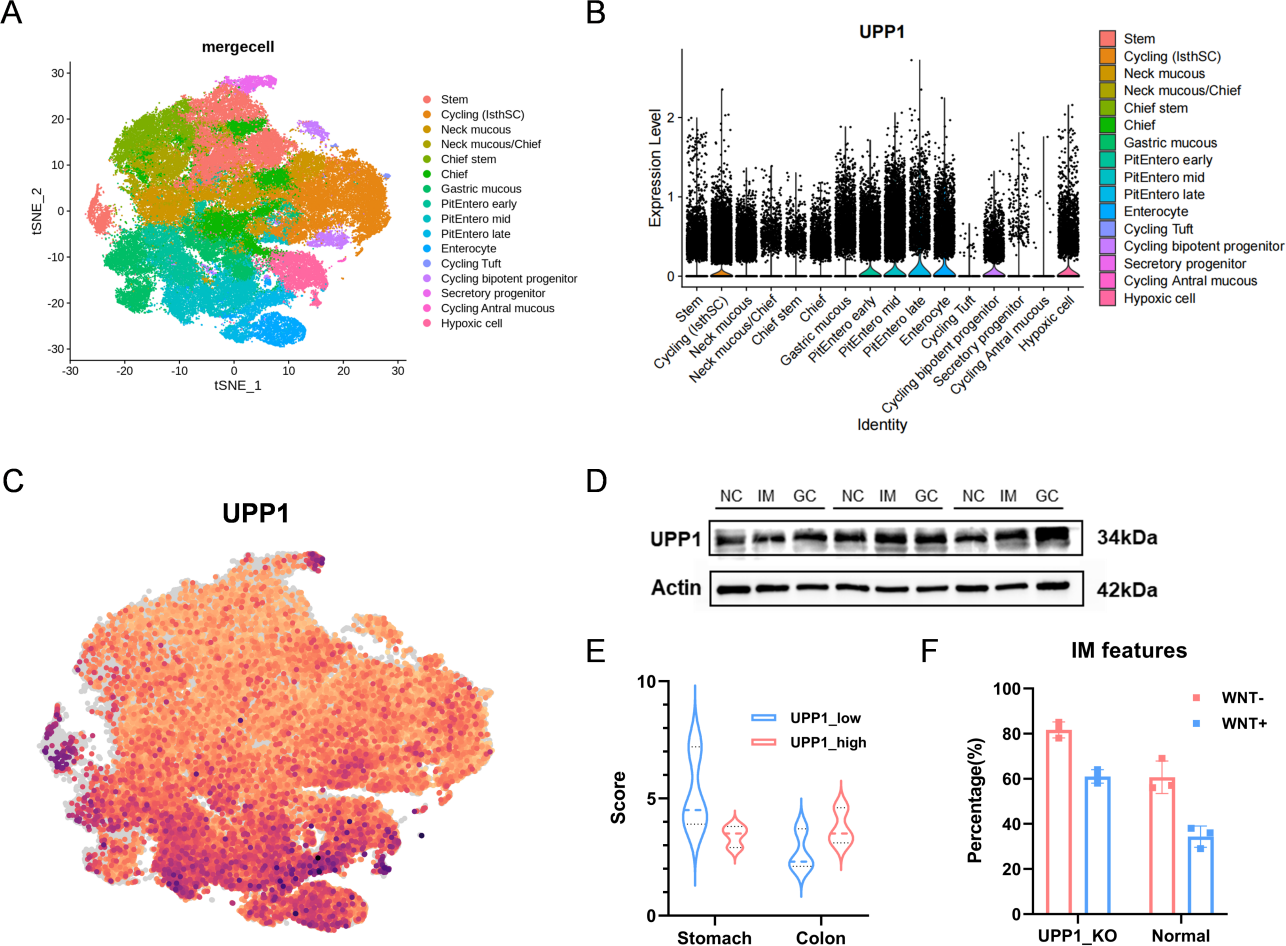
UPP1 promotes intestinal metaplasia in gastric epithelial organoid models. **(A)** Single-cell RNA-seq analysis of gastric organoids with initial epithelial clustering and annotation. **(B)** UPP1 expression across epithelial subtypes, enriched in IM-like cells. **(C)** Feature plot showing UPP1 distribution in UMAP space. **(D)** Western blot analysis of UPP1 in patient-derived normal, IM, and gastric cancer tissues. **(E)** Gastric and intestinal scores for UPP1-high vs. UPP1-low cells. **(F)** Organoid experiments showing increased IM-like morphology under WNT-depleted conditions, further enhanced by UPP1 knockout.

Gastric organoids provide a physiologically relevant platform to study epithelial differentiation under controlled conditions. We analyzed a single-cell RNA-seq dataset derived from human gastric organoids (**Figure 7A**). Initial clustering and annotation of epithelial subpopulations (marker genes shown in **Supplementary Table S2**) revealed distinct normal, intestinal metaplasia (IM)-like, and other lineage states. We next examined UPP1 expression across epithelial subtypes (**Figure 7B**). UPP1 was markedly enriched in IM-like cells compared with normal epithelial clusters, consistent with our earlier findings from tissue-based scRNA-seq and spatial transcriptomics analyses. Feature plot visualization confirmed that UPP1 expression was significantly enriched in IM cell populations, including early, mid, and late IM subclusters, compared with normal epithelial cells. This enrichment pattern highlights UPP1 as a transcriptional feature associated with the stepwise progression of intestinal metaplasia (**Figure 7C**). To validate these observations at the protein level, we collected gastric epithelial tissues from patients with normal mucosa, IM, and gastric cancer, each classified independently by at least three certified pathologists. Western blotting demonstrated that UPP1 protein levels progressively increased from normal epithelium to IM and further to gastric cancer (**Figure 7D**). Based on UPP1 expression levels, epithelial cells were stratified into UPP1-high and UPP1-low groups. We then calculated “gastric scores” and “intestinal scores” for each group using curated gene sets from the Human Protein Atlas(**Supplementary Table S3**). UPP1-low cells exhibited significantly higher gastric scores, whereas UPP1-high cells displayed elevated intestinal scores (**Figure 7E**), indicating that UPP1 serves as a marker of intestinal-type differentiation. Building on WNT pathway modulation, we further investigated the role of UPP1 as a potential mediator influencing the progression of gastric epithelial intestinal metaplasia. In organoid cultures without WNT3A supplementation, a significantly higher proportion of organoids developed IM-like morphology. Strikingly, UPP1 knockout in these cultures further increased the proportion of IM-like organoids compared to UPP1-intact controls (**Figure 7F**), confirming that UPP1 acts as a pro-intestinal metaplasia factor in the differentiation trajectory of gastric epithelial organoids.

### Helicobacter pylori infection alters gastric epithelial differentiation trajectory and induces UPP1 expression

**Figure 8:**
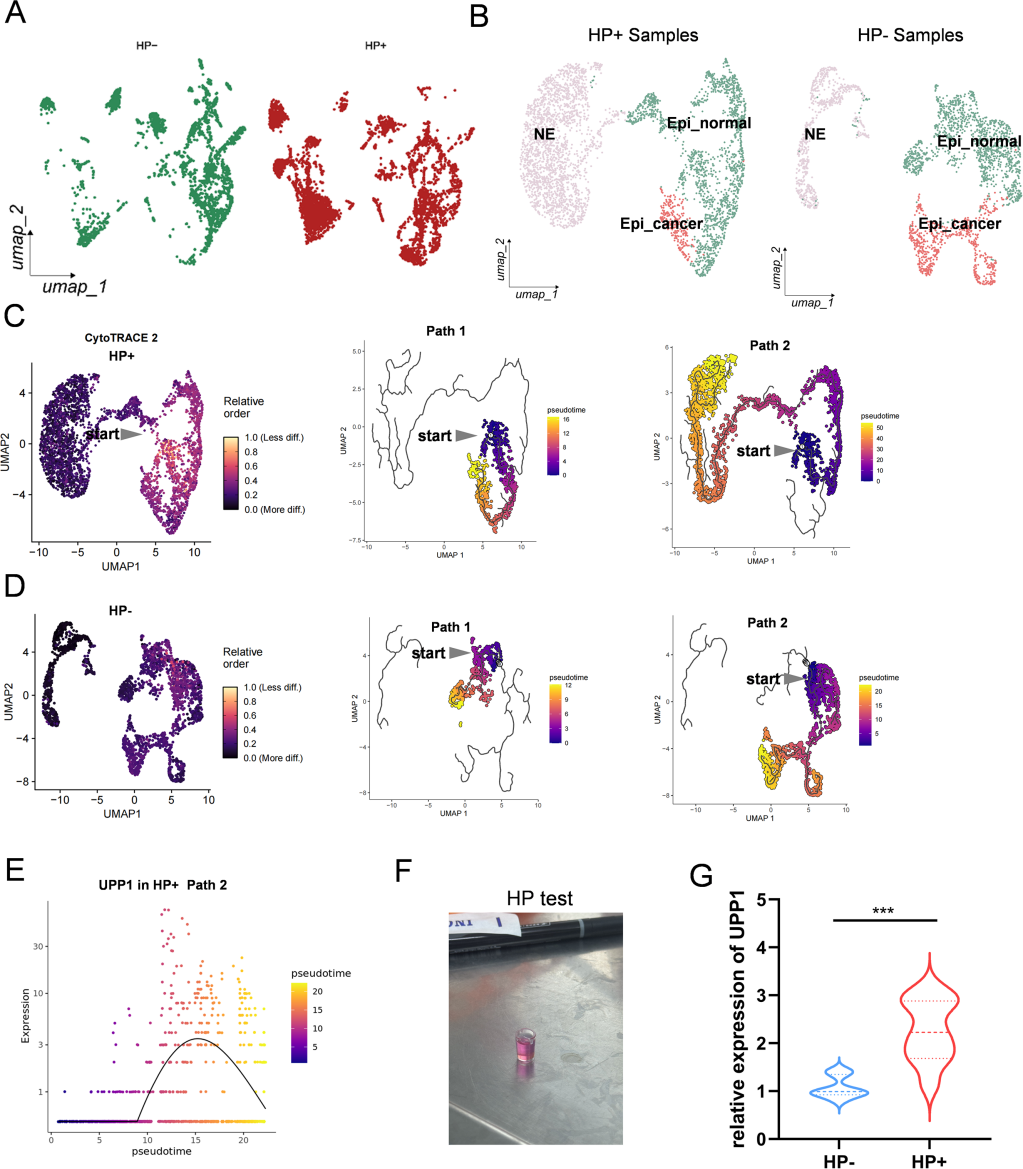
Helicobacter pylori infection alters gastric epithelial differentiation trajectory and induces UPP1 expression. **(A)** UMAP of epithelial cells from HP^+^ and HP^-^ samples. **(B)** Cell-type annotations of HP^+^ and HP^-^ epithelial populations, including NE cells, normal epithelial cells, and cancer-like epithelial cells. **(C)** HP^+^ pseudotime trajectory analysis identifying two differentiation paths: toward NE and toward cancer-like epithelium. **(D)** HP^-^ pseudotime trajectories for comparison. **(E)** UPP1 expression along the malignant differentiation path in HP^+^ samples. **(F)** Rapid urease test confirming HP infection in clinical specimens. **(G)** qPCR validation showing higher UPP1 expression in HP^+^ tissues compared with HP^-^ tissues. ***p < 0.001.

To further explore the role of HP infection in shaping gastric epithelial fate decisions, we stratified single cell data from epithelial compartments into HP^+^ and HP^-^ groups (**Figure 8A**). HP^+^ and HP^-^ samples each contained a broad spectrum of epithelial subpopulations, indicating that infection status spans across epithelial heterogeneity. We performed separate cell-type annotations for HP^+^ and HP^-^ samples (**Figure 8B**). Epithelial cells were subclassified into neuroendocrine (NE) cells, normal epithelial (Epi_normal), and cancer-like epithelial (Epi_cancer) cells. This allowed the reconstruction of distinct lineage trajectories conditioned on infection status. In HP^+^ epithelial cells, we applied CytoTRACE and Monocle3 to infer differentiation trajectories (**Figure 8C**). All clustering and annotation details are provided in **Supplementary Figure S4**. Two major pseudotime branches emerged from a common progenitor origin: one progressing toward the NE lineage, and the other toward cancer-like epithelial cells. Notably, Path 2, which leads to malignant epithelial states, displayed increasing pseudotime values and progressive transcriptional reprogramming. To compare the effect of infection, we performed similar trajectory analysis on HP^-^ epithelial cells (**Figure 8D**). Although a similar bifurcating pattern was observed, the degree of differentiation and pseudotime progression appeared attenuated in HP^-^ samples, suggesting that HP infection accelerates the epithelial transition toward oncogenic states.

Focusing on UPP1, we examined its expression dynamics along the malignant differentiation path in HP^+^ samples. UPP1 expression markedly increased along Path 2, peaking at late pseudotime stages (**Figure 8E**), consistent with previous findings in pseudotime, spatial transcriptomics, and organoid models. Although a similar bifurcating pattern was observed, the degree of differentiation and pseudotime progression appeared attenuated in HP^-^ samples, suggesting that HP infection accelerates the epithelial transition toward oncogenic states. Based on the differentiation trajectory toward tumor states in the HP^+^ group (Path 1), we performed pseudotime expression analysis of key genes. Tumor markers CEACAM5 and CEACAM6 were found to increase along the transition from normal epithelium to cancer-like epithelium, whereas the epithelial marker MUC5AC decreased during this process. In contrast, MUC6 showed an upward trend during tumor-like differentiation (**Supplementary Figure S5**). Moreover, we summarized the top-ranked genes with dynamic changes along pseudotime and identified PGC as significantly altered during the transition from normal to malignant states, highlighting a close association between chief cells and epithelial carcinogenesis. To validate the impact of HP infection on UPP1 expression in clinical specimens, we collected gastric epithelial tissues and performed the rapid urease test to determine HP infection status (**Figure 8F**). Subsequent qPCR analysis showed significantly elevated UPP1 mRNA levels in HP^+^ tissues compared to HP^-^ counterparts (**Figure 8G**), confirming that HP infection directly promotes UPP1 upregulation *in vivo*. In conclusion, these data demonstrate that HP infection reprograms epithelial differentiation trajectories, promoting a shift toward tumor-like epithelial states and upregulating UPP1 expression, supporting its role as a critical mediator of HP^-^ driven gastric carcinogenesis.

## Discussion

In summary, by integrating single-cell RNA sequencing, spatial transcriptomics, and functional validation, our study delineates the cellular and molecular architecture of gastric epithelial lineage progression, spanning normal mucosa, intestinal metaplasia (IM), and carcinoma, with particular emphasis on HP-associated transformation. In gastric glands, stem cells located in the isthmus and basal regions serve as the origin of epithelial differentiation, possessing distinct potentials to give rise to either normal or intestinal-type epithelium (17, 18). Understanding the factors that determine these divergent differentiation trajectories is therefore of central interest. Our analyses demonstrated sustained WNT pathway activation in metaplastic and tumorigenic regions, alongside NF-κB pathway activation in HP-associated inflammation (19, 20). Among differentially expressed genes, UPP1 emerged as a consistent and robust driver of intestinal-type differentiation, showing progressive upregulation along pseudotime trajectories toward cancer-like epithelium in both HP^+^ and HP^-^ contexts, but particularly accentuated in HP^+^ samples. Functional validation using gastric organoids confirmed that UPP1 promotes metaplastic phenotypes, especially under WNT-depleted conditions, and that its knockdown reduces proliferative and invasive capacities in gastric cancer cell lines. Clinical validation demonstrated that UPP1 is elevated in GC compared to normal mucosa, correlates with advanced TNM stage, and predicts poorer overall survival, establishing it as both a mechanistic mediator and a potential prognostic biomarker in gastric carcinogenesis.

Our findings resonate with and extend current international research. A large number of studies on early-stage gastric cancer and large-scale longitudinal cohort studies have shown that IM is a critical turning point in the Correa cascade, with its extent and histological subtype strongly predicting progression to early gastric cancer (21–23). Both regions have emphasized HP eradication as a primary prevention strategy, yet clinical data indicate that a substantial subset of patients still progress to GC despite successful eradication, underscoring the need for molecular biomarkers to identify high-risk individuals (24, 25). Numerous oncology studies have emphasized the importance of integrating histological surveillance with molecular profiling, underscoring its value for risk stratification, early detection, and individualized patient management. This approach not only enhances diagnostic precision but also provides insights into tumor evolution and therapeutic responsiveness (26–30). Our identification of UPP1 as a driver linking inflammation, WNT signaling, and epithelial reprogramming aligns with this unmet need, offering a molecular bridge between epidemiological risk factors and cellular-level transformation.

Nevertheless, our study has limitations that warrant consideration. First, while scRNA-seq and spatial transcriptomics offer unprecedented resolution, our dataset is cross-sectional; hence, it cannot fully capture the temporal dynamics of IM progression *in vivo*. Longitudinal sampling from the same patients before and after HP eradication, as has been attempted in Japanese endoscopic surveillance programs, would be valuable for validating our trajectory models. Second, our organoid cultures, though physiologically relevant, lack the complexity of the *in vivo* gastric microenvironment, particularly stromal, immune, and microbial interactions, which have been shown in murine models to profoundly influence epithelial fate. Third, the precise molecular circuitry connecting UPP1 to downstream effectors—whether through direct modulation of pyrimidine metabolism (31–33), interaction with WNT transcriptional targets, or cross-regulation with NF-κB signaling mdash;remains to be elucidated. Dissecting these connections will be essential to move from correlative observations to causal understanding.

From a broader perspective, the role of UPP1 in gastric epithelial transformation raises several compelling research questions. Given its enzymatic function in pyrimidine metabolism, it is plausible that UPP1-mediated metabolic rewiring could provide the biosynthetic advantage necessary for rapidly proliferating metaplastic or malignant cells. Similar metabolic–differentiation links have been reported in colorectal cancer and pancreatic intraepithelial neoplasia, suggesting a conserved oncogenic principle that warrants cross-organ investigation (31, 34). Moreover, the interplay between UPP1 and WNT signaling suggests that metabolic and developmental pathways may be co-opted in parallel to drive epithelial plasticity. This is in line with recent single-cell studies from Korean cohorts showing that WNT-high metaplastic glands exhibit distinct metabolic signatures compared to WNT-low counterparts.

Looking ahead, several avenues could further advance this work. The integration of high-dimensional single-cell and spatial profiling with *in situ* lineage tracing in genetically engineered mouse models could provide causal evidence for UPP1’s role in IM-to-GC progression. Development of patient-derived organoid–immune co-culture systems would allow interrogation of how immune–epithelial crosstalk influences UPP1 activity, particularly in the context of HP persistence versus eradication. From a translational perspective, the identification of small-molecule inhibitors, RNA interference strategies, or targeted protein degradation systems against UPP1 could open new therapeutic possibilities. Such interventions could be particularly powerful when combined with WNT pathway modulation or post-eradication surveillance in patients with high-risk IM.

In conclusion, our study not only identifies UPP1 as a pivotal molecular driver linking HP-induced inflammation and WNT-mediated epithelial reprogramming, but also provides a methodological framework for integrating single-cell, spatial, and functional analyses to uncover actionable targets in precancerous gastric lesions. In the evolving landscape of gastric cancer prevention, such integrated approaches are poised to refine risk stratification, inform targeted surveillance, and ultimately enable earlier and more effective intervention in the precancerous stages of gastric carcinogenesis.

## Data Availability

The original contributions presented in the study are included in the article/**Supplementary Material**. Further inquiries can be directed to the corresponding authors.
