# Supplementary material for: Integrated single-cell and spatial transcriptomics reveal the differentiation drivers of gastric epithelial lineage progression
Source: Front Immunol. 2026 Feb 9;17:1712830. doi: 10.3389/fimmu.2026.1712830 (PMC12926446; doi:10.3389/fimmu.2026.1712830)
Supplement: Supplementary file 1 [file DataSheet1.pdf]

Supplementary figure

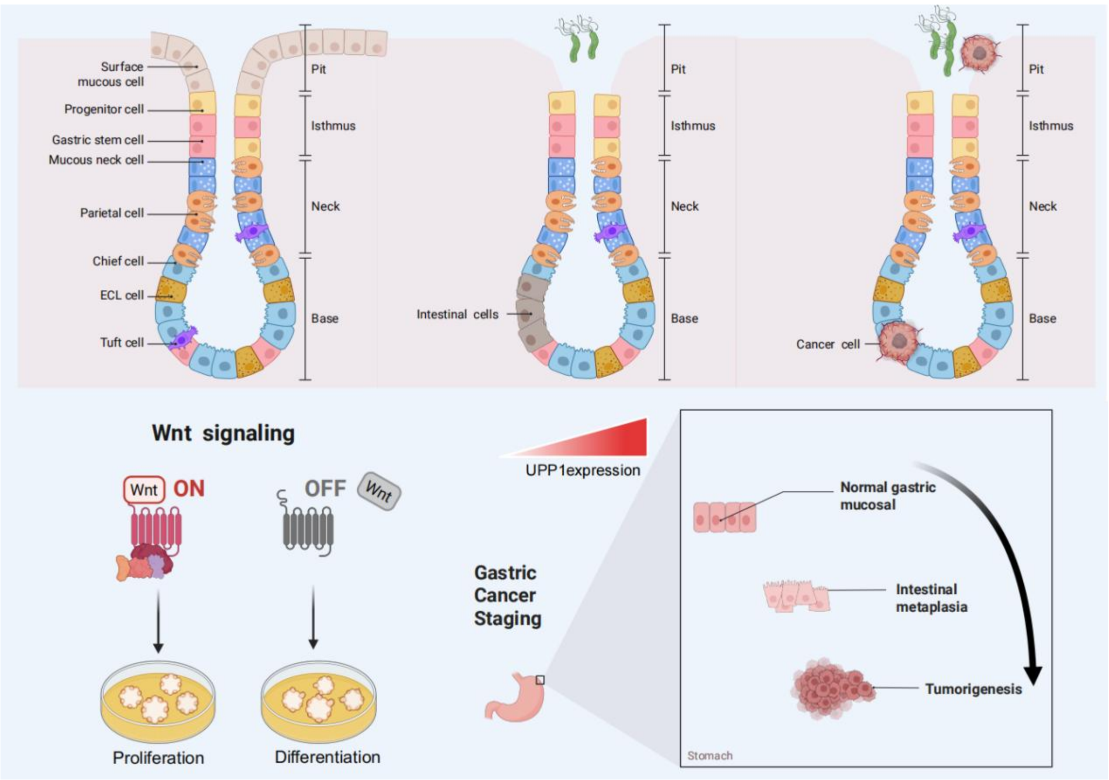

Graphical Abstract.
Integrated single-cell and spatial transcriptomics identify UPP1 as a key driver linking *Helicobacter pylori*–induced inflammation, WNT-mediated epithelial reprogramming, and gastric carcinogenesis.

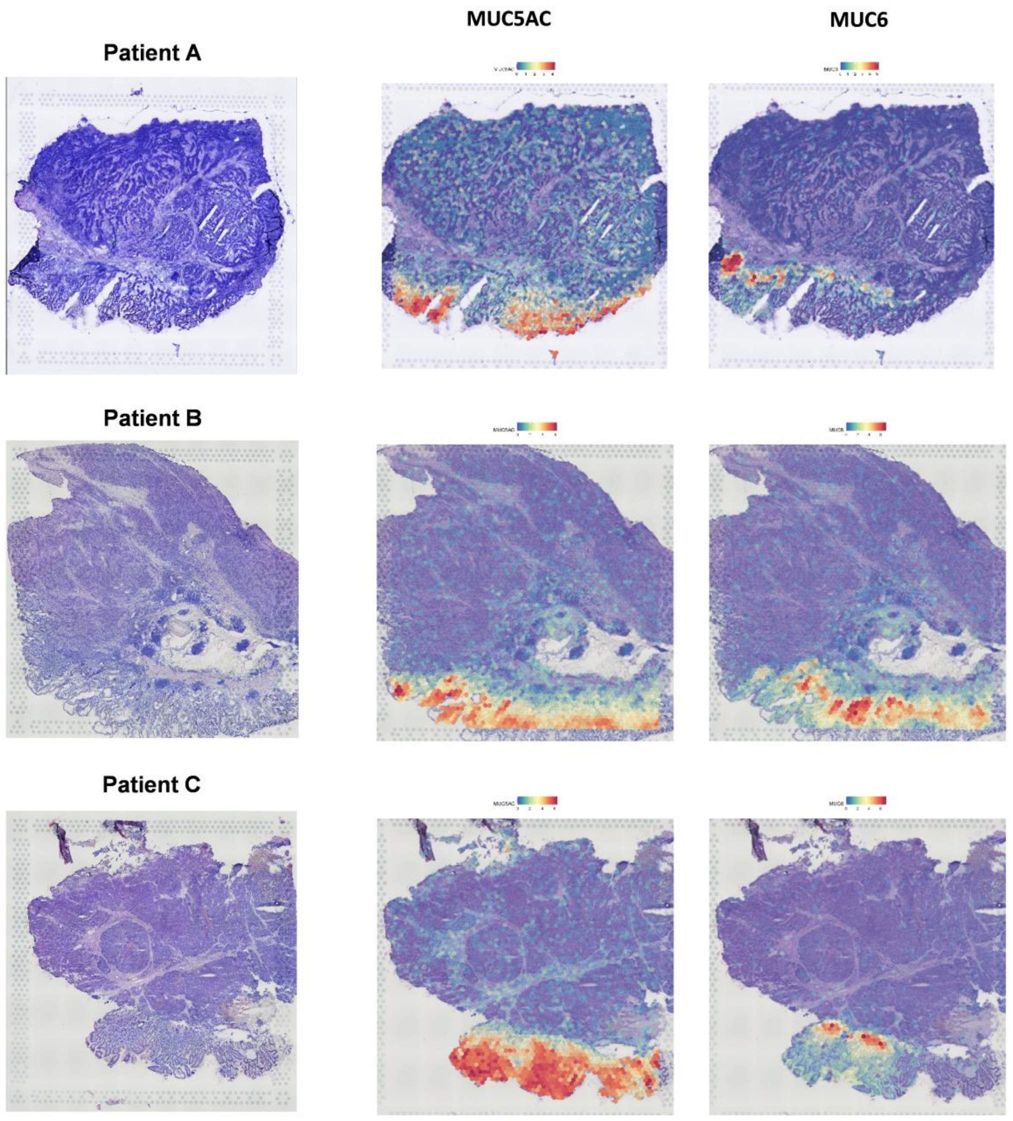

Supplementary Figure 1. Spatial transcriptomics showing the localization of gastric epithelial markers MUC5AC and MUC6 across multiple gastric tissue sections.

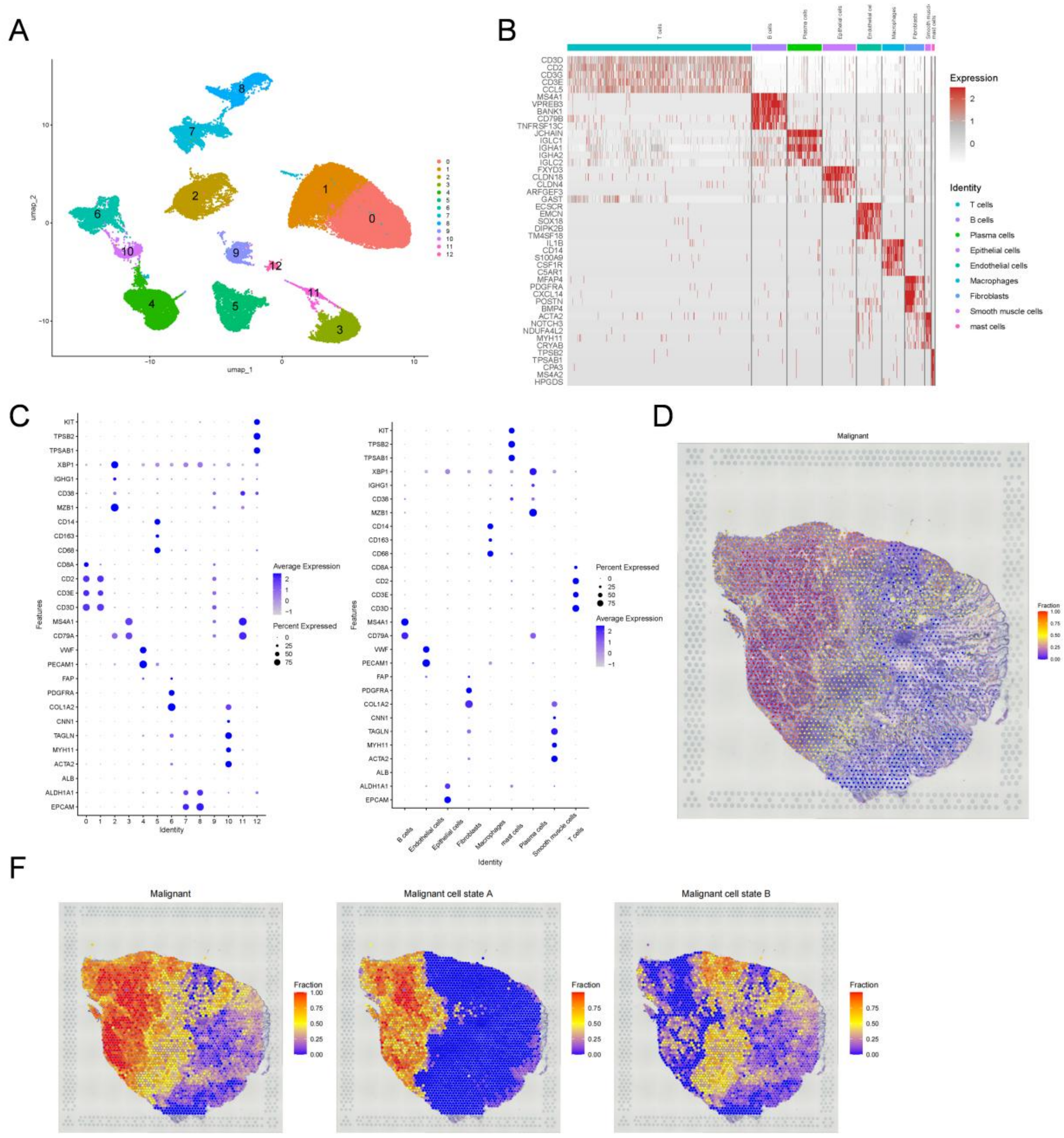

Supplementary Figure 2. Single-cell clustering with canonical markers and spatial transcriptomics–based regional definition of gastric tissues.

(A) UMAP visualization showing clustering of all single cells.

(B) Heatmap displaying the expression of representative marker genes for each identified cell cluster.

(C) Dot plot showing the expression of canonical gastric marker genes across different clusters.

(D) Spatial transcriptomics map showing the distribution of spot-level gene expression across gastric tissue sections.

(F) Identification of tumor regions within gastric tissue based on spatial transcriptomics data.

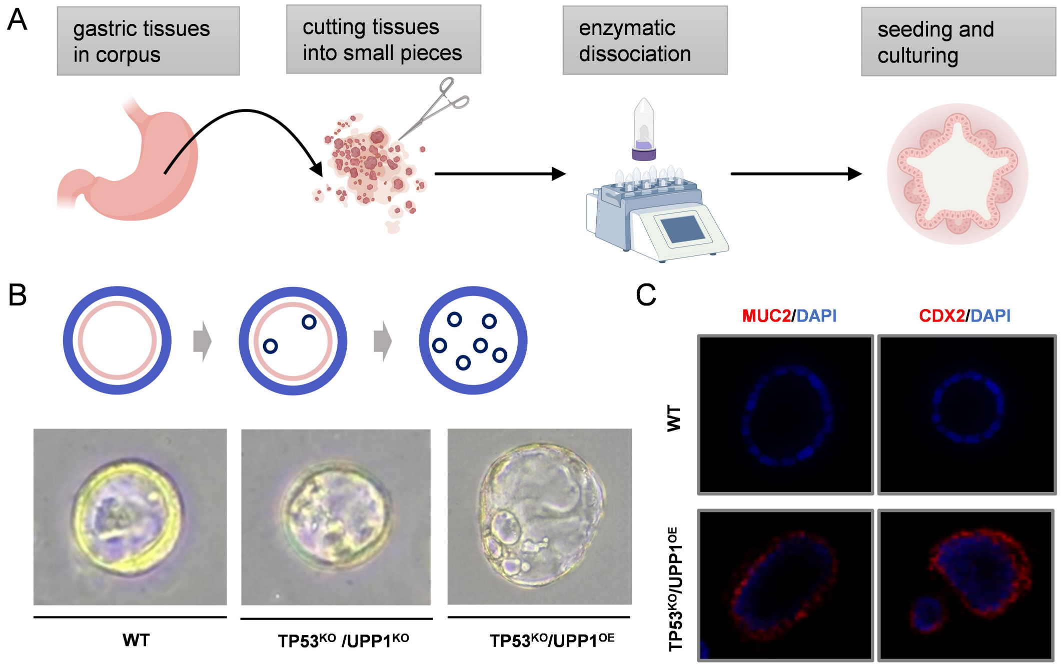

Supplementary Figure 3. Workflow of gastric organoid culture and functional assessment showing that UPP1 knockout promotes intestinal metaplasia–like phenotypes, whereas UPP1 overexpression suppresses intestinal differentiation.

(A) Schematic illustration of the workflow for gastric organoid isolation and culture.

(B) Representative images showing morphological changes of gastric organoids under UPP1 knockout or UPP1 overexpression, exhibiting distinct intestinal metaplasia–like features.

(C) Representative images showing increased expression of intestinal marker genes in gastric organoids upon UPP1 overexpression.

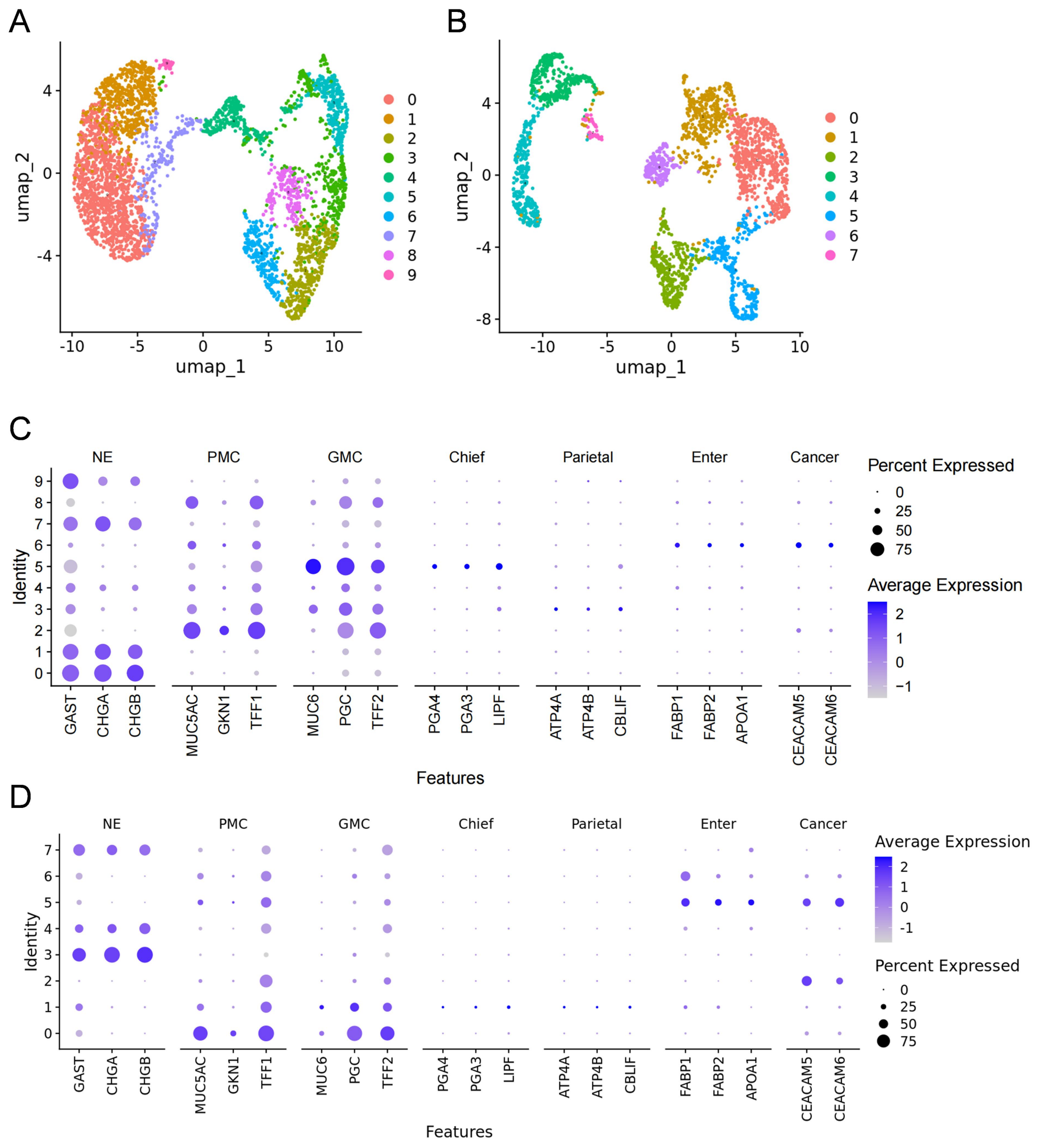

Supplementary Figure 4. Re-annotation of epithelial cells from Helicobacter pylori (HP)–positive and HP–negative samples with validation by canonical marker genes.

(A) UMAP visualization showing clustering of epithelial cells from Helicobacter pylori–positive samples.

(B) UMAP visualization showing clustering of epithelial cells from Helicobacter pylori–negative samples.

(C) Dot plot showing the expression of key marker genes in epithelial clusters from Helicobacter pylori–positive samples.

(D) Dot plot showing the expression of key marker genes in epithelial clusters from Helicobacter pylori–negative samples.

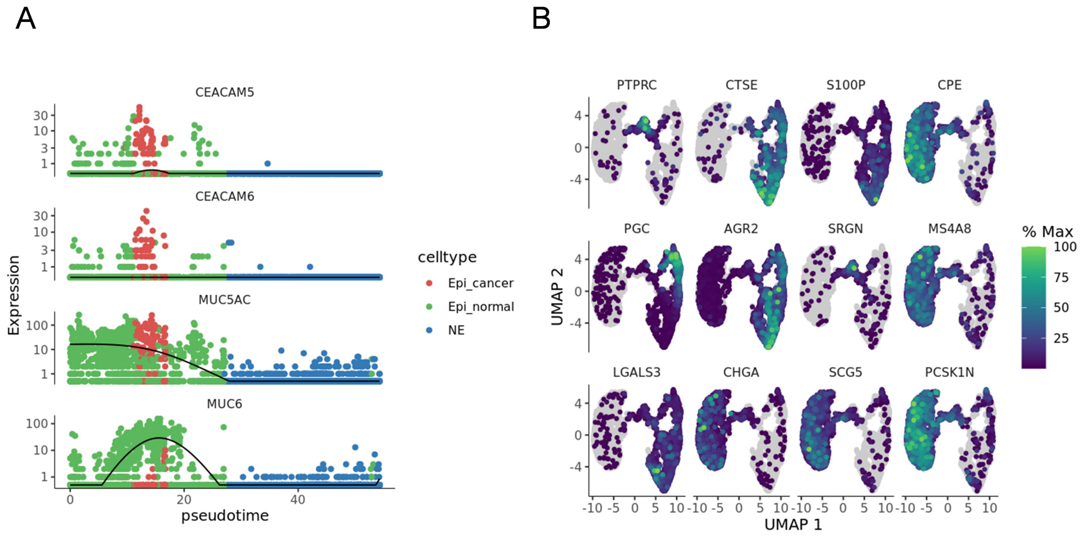

Supplementary Figure 5. Expression dynamics of key genes along the differentiation trajectory in Helicobacter pylori–positive epithelial cells.

(A) Expression changes of key oncogenic and epithelial marker genes along the differentiation trajectory of Helicobacter pylori–positive epithelial cells.

(B) Expression patterns of the most dynamically regulated genes along the same differentiation trajectory.
